# Improved Prediction Model of Protein and Peptide Toxicity by Integrating Channel Attention into a Convolutional Neural Network and Gated Recurrent Units

**DOI:** 10.1021/acsomega.2c05881

**Published:** 2022-10-27

**Authors:** Zhengyun Zhao, Jingyu Gui, Anqi Yao, Nguyen Quoc Khanh Le, Matthew Chin Heng Chua

**Affiliations:** † Institute of Systems Science, National University of Singapore, 25 Heng Mui Keng Terrace, Singapore 119615, Singapore; ‡ Professional Master Program in Artificial Intelligence in Medicine, College of Medicine, Taipei Medical University, Taipei 106, Taiwan; § Research Center for Artificial Intelligence in Medicine, Taipei Medical University, Taipei 106, Taiwan; ∥ Translational Imaging Research Center, Taipei Medical University Hospital, Taipei 110, Taiwan

## Abstract

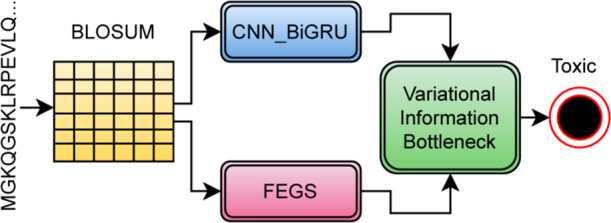

In recent times, the importance of peptides in the biomedical domain has received increasing concern in terms of their effect on multiple disease treatments. However, before successful large-scale implementation in the industry, accurate identification of peptide toxicity is a vital prerequisite. The existing computational methods have reached good results from toxicity prediction, and we present an improved model based on different deep learning architectures. The modification mainly focuses on two aspects: sequence encoding and variational information bottlenecks. Consequently, one of our modified plans shows an obvious increase in sensitivity, while the rest show good performance meanwhile adding novelty in the peptide toxicity prediction domain. In detail, our best model could achieve an accuracy of 97.38 and 95.03% in protein and peptide toxicity predictions, respectively. The performance was superior to previous predictors on the same datasets.

## Introduction

Peptide-based therapeutics have been the hot spot of the biomedical industry for half a century.^1,2^ Compared to small molecules, the properties of peptides such as high specificity, high penetration, and ease of production make them outstanding. The toxicity of peptides and proteins, however, is one of the barriers which stops them from large-scale applications. On a positive note, the known toxicity of peptides has been increasing with human research, and because of that, scientists have proposed various multiple-recognition approaches based on those contributions.^3,4^

Presently, mainstream research in this field is based on biological similarities or machine learning. Traditionally, similarity-based methods such as the basic local alignment search tool (BLAST)^5^ measure the resemblance of pairwise sequences on a local and global scale using alignment search or infer sequence toxicity from an analogous sequence. However, this approach has several drawbacks. First, the peptide of interest must have an analogous toxic peptide. Then, the outcome experiences a harsh dive as the data scale becomes large. Lastly, e-value cutoffs and arbitrary sequence similarities must be customized; meanwhile, prediction outcomes are heavily determined by it. Different from methods that originated from biology similarity, methods from machine learning fields pay attention to collecting differentiated information related to toxicity while concluding peptide toxicity with samples marked as positive and nonpositive. The ClanTox model^6^ analyzes animal venom using 545-dimensional traits derived from origin sequences and an augmented stump classifier. ToxinPred^7^ identifies toxic and nontoxic peptides by using a combination of support vector machine and statistical features of peptide sequences. However, the above machine learning methods dismissed the deep features of amino acids within the sequence. Deep learning methods therefore were proposed to extract more discriminative features and achieved better performance in prediction.^8,9^ In ToxDL,^4^ the model employs both sequence information and protein information in biology but is generic and lacks customization, and it needs to search specific protein domains for embeddings. In ATSE,^10^ the position-specific scoring matrices must be searched using PSI-BLAST^11^ in a big database, thus causing low efficiency. Furthermore, when searching in different scale databases, different results will be achieved. To solve these drawbacks, ToxIBTL, which mainly implements transfer learning and information bottleneck methodologies,^3^ was proposed recently to effectively make peptide and protein toxicity predictions.

To improve statistical confidence in predicted toxic peptides, a novel method is required to reduce the number of misclassified samples. Under such a background, our key objective is to improve the predictive performance compared to other previous studies on the same problem. Throughout this study, we briefly discuss five representative modification designs attempted and analyze their performances on protein and peptide datasets, respectively. The five modification designs were based on the transformer-based model that has been applied successfully in molecular science^12,13^ and can be summarized as follows:

1. Applied self-attention to weigh the hidden state of the bidirectional gated recurrent unit (BiGRU) network to enhance the feature extraction capabilities of the convolutional neural network (CNN) and BiGRU (CNN_BiGRU).
2. Replaced the BiGRU with an innovative model—transformer (multihead attention as the basis) to enable parallel computations while maintaining strong abilities to extract hidden components in evolutionary information extracted from the BLOSUM62 matrix.
3. Applied channel attention to CNN and BiGRU in order to improve feature extraction capabilities and reduce the feature vector dimension.
4. Incorporated numerical information into the feature extraction based on graphical and statistical feature (FEGS) encoding^14^ process to take advantage of the AAIndex1 dataset^15^ and extract the physicochemical information more precisely.
5. Modified information bottleneck (a method used in ToxIBTL paper^3^) to deep variational information bottleneck^16^ for better calculation of compressed sequence representations.

## Materials and Methods

### Benchmark Datasets

We used identical protein and peptide datasets with the benchmark paper to evaluate the performance of optimization designs. Therefore, the models were built on the protein dataset from Pan et al.^4^ This dataset mainly contains 4472 toxic protein sequences and 6341 nontoxic sequences. In addition to protein level prediction, we also had another model for peptide level prediction, and this model utilized the dataset from Wei et al.,^3^ which consists of 3864 sequences. Both datasets used the same FASTA format which is popular for representing biological sequences. In these datasets, all sequences did not share any similarity greater than 40% to ensure that the model will not rely too much on overestimation or overfitting. The benchmark studies also separated the data into training and testing; thus, we also used the same data proportion in our study. The training data was used to build the models via 10-fold cross-validation (in which we divided the dataset into 10 folds and each fold served as internal validation data one time). On the other hand, the testing data was used as external validation data to evaluate the performance of models. Table 1 displays the statistical details of our referenced datasets.

**Table 1:** Overview of the Benchmark Dataset

	dataset	positive data	negative data
protein	training	4413	5671
	testing	59	670
peptide	training	1642	1642
	testing	290	290

### Sequence Encoding

We followed the encoding method from the ToxlBTL model architecture^3^ which mainly consists of three steps. First, CNN_BiGRU captured hidden information on the local and global scale with the aid of evolutionary profiles converted from raw sequences; in the meantime, the FEGS model^14^ used unconverted sequences to obtain properties from graphics (a graphical representation of protein sequences) and statistics. Second, the evolutionary and physicochemical feature vectors were concatenated and an information bottleneck was used to optimize the concatenated features. Third, predictions of whether the sequence was toxic were made based on the above-mentioned features.

To extract the potential differentiated features hidden in evolutionary information, the BLOcks SUbstitution Matrix (BLOSUM) grid^17^ was fed into a two-dimensional CNN layer to determine the correlation between amino acids on a local scale. There are different types of BLOSUM matrices, and this study used BLOSUM62 which is similar to the ToxIBTL model.^3^ Since all sequences have different lengths, we also used the same approach as Pan et al.^4^ to truncate the long sequences to a maximum length of 1002 as well as add padding to short sequences. The output of the CNN is then fed into a BiGRU layer to derive long and short dependency information between the extracted local correlation and captured sequence-order effects. In this way, we can obtain a 2048-dimensional feature vector to represent the sequence. To better represent each sequence to encompass the perspective of its biophysical and biochemical properties, another feature extraction method FEGS^14^ is introduced to extract the graphical and statistical features of peptide and protein sequences. In this step, we can obtain a 578-dimensional feature vector to represent the sequence.

CNN_BiGRU provides properties in evolution, and FEGS provides properties in physiochemistry. These properties are linearly blended, and the information bottleneck principle was implemented to squeeze the combined features afterward. Following this, the optimized feature was forwarded into the fully connected layer, and a sigmoid layer was employed to perform categorization.

### Applied Self-Attention in CNN_BiGRU

**Figure 1:**
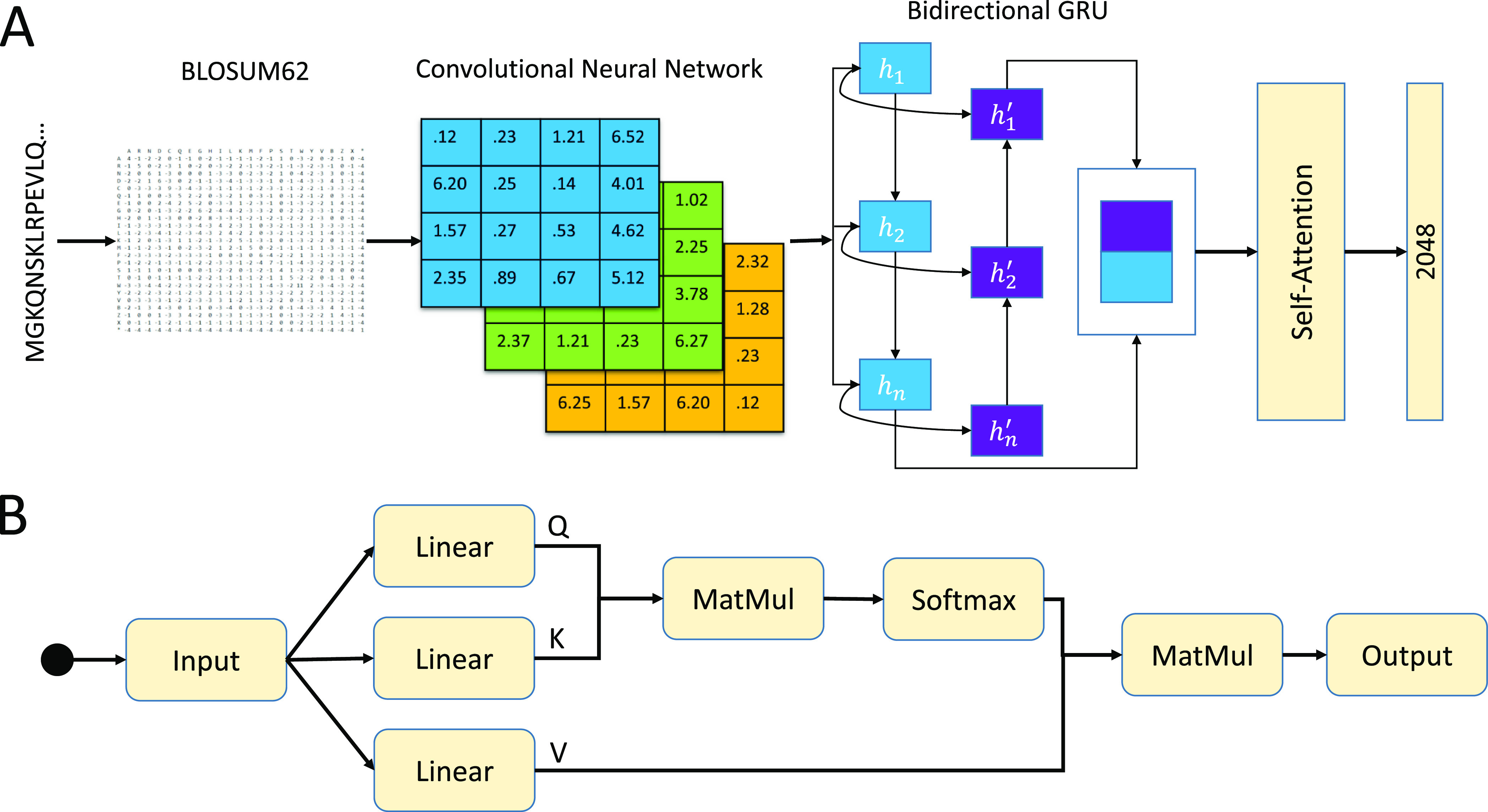
Revised architecture of modification design #1. (A) CNN_BiGRU + self-attention; (B) self-attention calculation process.

We applied different optimized techniques to the model implementation to reach optimal performance. The first modification design is to add self-attention to the BiGRU network to enhance the feature extraction capabilities of CNN_BiGRU. In the original model architecture, the raw sequences are converted to evolutionary profiles, and then latent information on a local and global scale was captured after going through CNN_BiGRU. All the information contributes equally during the computation of the BiGRU network. Due to the fact that some information is likely to play a more important role than others in predicting peptide toxicity, self-attention mechanisms may assign different weights to the information captured by the hidden layer. The revised model architecture of CNN_BiGRU is shown in Figure 1. In the revised architecture, the raw sequences were still converted to evolutionary profiles and sent into the compound network to obtain the 2048 dimensional features. Then, we added a self-attention layer to facilitate the interaction between input vectors and find out which should be paid more attention to (“attention”). Interactions and attention scores together constitute the final outputs.
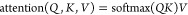
1where *Q* = query, *K* = key, and *V* = value. In practice, the process of self-attention can be described as query mapping with the result of a set of key-value pairs, where the keys, values, query, and outputs are all vectors. The output is computed as a weighted sum of the values, where the weight assigned to each value is computed by a compatibility function of the query with the corresponding key. The two most commonly used attention functions are dot-product (multiplicative) attention and additive attention. Here, we use the former because it is more efficient in time and space consumption. The calculation formula of dot-product attention is also shown in Figure 1.

### Replaced BiGRU with a Transformer (Based on Multihead Attention)

**Figure 2:**
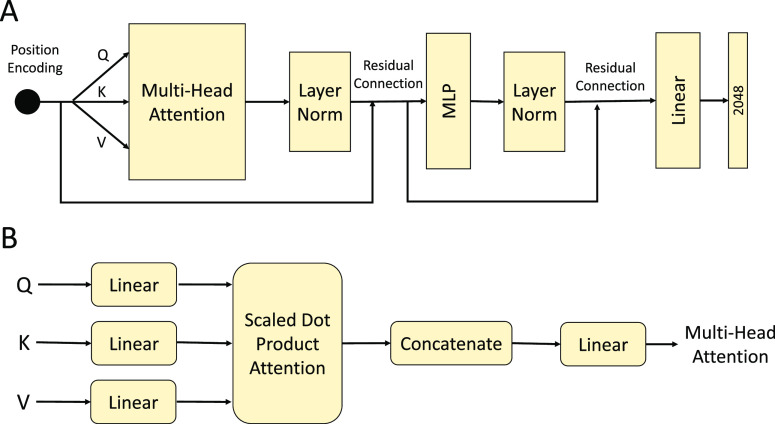
Revised architecture of modification design #2. (A) Replace BiGRU with transformer and (B) multihead attention calculation process.

Upon considering the possible causes for the unsatisfactory performance of the first modification design, we came up with the idea of replacing BiGRU with a transformer, which is based solely on attention mechanisms, to depict dependencies between the input and output on a global scale. Figure 2 shows the corresponding model architecture. To extract local correlation between amino acids through the local perceptual domain, the revised workflow feeds the BLOSUM62 matrix^17^ of a protein or peptide sequence into a two-dimensional convolutional layer with a nonlinear activation function. Thereafter, the transformer encoder block uses the convolutional layer outcome to achieve a sequence of continuous representations. Since no recurrent and convolutional structures are included in the transformer model, in order for the model to make use of the order of the sequence, “positional encoding” is used to provide information about the token position in the sequence. A positional encoding can be summed with an embedding by using the same dimension *d*. The encoder constitutes of six layers, with each layer having two sublayers, simply put as a multihead self-attention structure followed by a simple fully connected feed-forward network. The layers are normalized after residual connections are applied to each sublayer.^18^ That is, the output of each sublayer is LayerNorm(*x* + Sublayer(*x*)), where Sublayer(*x*) is the function implemented by the sublayer itself.

When the multihead self-attention model is running, there are eight scaled dot-product attention layers working concurrently. Here, we linearly project the queries, keys, and values eight times with different, learned linear projections to *dk*, *dk*, and *dv* dimensions, respectively. On each of these projected versions of queries, keys, and values, we then performed the attention function in parallel, yielding *dv*-dimensional output values. These are concatenated and once again projected, resulting in the final values, as depicted in Figure 2B. The model is able to cooperatively attend to information from different representation subspaces at different positions by using multihead attention. With a single attention head, averaging inhibits this. In this work, we employed *h* = 8 parallel attention layers, and for each of these, we use *dk* = *dv* = *d*/*h* = 64.
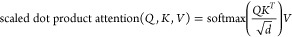
2
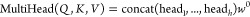
3where
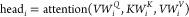
4In addition to attention sublayers, a fully connected feed-forward network (MLP), which has a rectified linear unit (ReLU) function associating two fully connected layers, is within every encoder layer. The linear transformations have an identical mechanism; however, the number of dimensions are varied across layers. For example, the parameter in the encoder layer is 512, while the parameter in the inner layer is 2048.
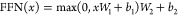
5

### Applied Channel Attention in CNN_BiGRU

**Figure 3:**
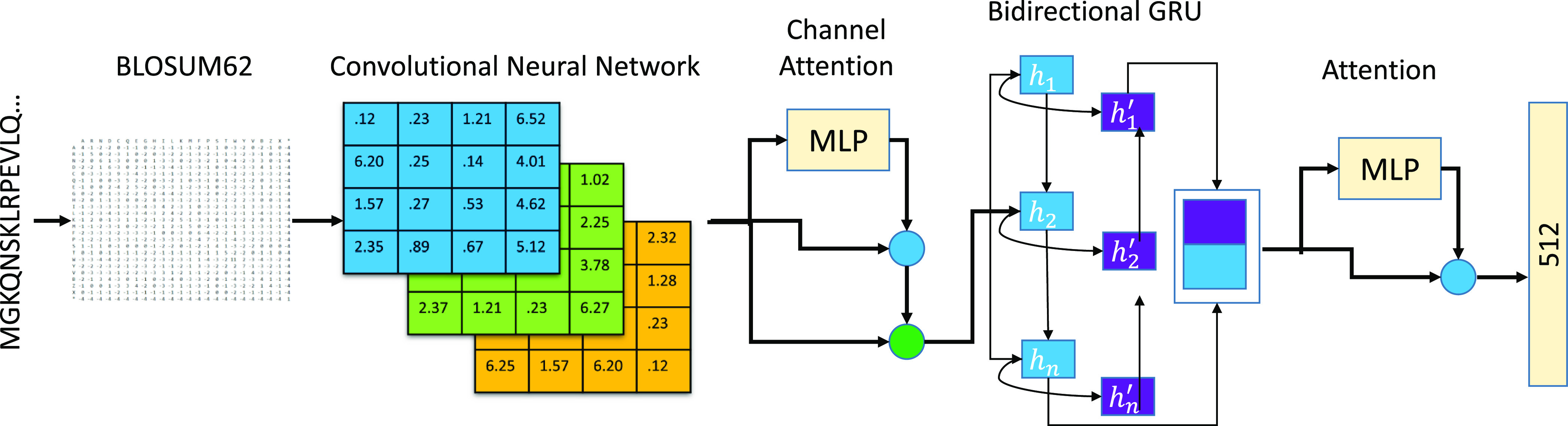
Revised architecture of modification design #3 (CNN_BiGRU + channel attention).

As the first two modifications are based on self-attention, another alternative is to add channel attention to the CNN and BiGRU framework. The performance of CNNs can be dramatically improved with the integration of channel attention, which only involves a few parameters and can result in significant performance improvements. Because of feature map channels’ property of feature identification, channel attention is another method to identify what is meaningful by squeezing the dimension of the input feature map dimensions in terms of space. Figure 3 shows the corresponding revised model architecture. The channel attention mechanism designed here consists of two parts. In the first step, MLP is used to obtain the channel dimension attention weight, then the attention weight is normalized with the sigmoid function, and then the normalized weight is broadcast to the original input, which consists of multiplication with the original input one by one to complete the weighted calibration by channel attention. In the second step, a similar approach was utilized to obtain the attention weights of the four hidden layers of the BiGRU network through MLP and perform a weighted summation that reduces the output feature to 512 dimensions.

### Added Numerical Information into FEGS

In the original FEGS model paper,^14^ the author mentioned two possible improvements. First, the structural information of protein is not a valid input for the current model. Second, the author wanted to include the values of the AAindex1^15^ for individual amino acids in each physicochemical property of amino acids to add some numerical information to FEGS. Two significant drawbacks were raised in introducing structure-related information. First, the used dataset only contained information about the primary structure, and most transcoding methods associated with the structure are dependent on data about the secondary structure. Second, most structure-related transcoding methods are based on the energy matrix between residues, which cannot be well combined with the existing two methods. Therefore, to improve the FEGS model, we mainly consider how to introduce numerical information.

**Figure 4:**
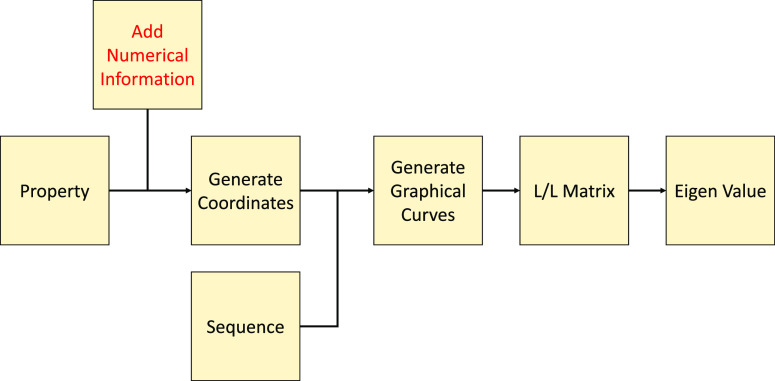
Revised architecture of modification design #4 (add numerical information into FEGS).

In a property, there is a corresponding AAIndex1 data for each amino acid to describe the physiological and chemical properties associated with that amino acid. All we need to do is use accurate numerical information for transcoding calculations. We redesigned the generation rules for 20 coordinates corresponding to each property, as shown in Figure 4. Based on AAIndex1 corresponding to each amino acid in each property, we sort the amino acids from small to large to form a sequence of length 20, then calculate the cumulative value of the first *i* amino acid sequences, and scale them from 1 to 20, denoted ζ. Based on the above-obtained series, we define the coordinates of each amino acid under each property as follows
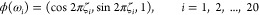
6

After getting the coordinates of each amino acid, we define the pairwise coordinates as
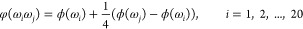
7We then transcode a certain sequence to obtain the spatial curve corresponding to the sequence and denote the sequence with *N* amino acids. The next step is to deduce its graphical curve corresponding to the property. Note that the initial point of the curve is, then the next coordinate is
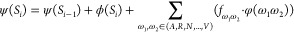
8In the above formula, *f* is the frequency of the amino acid pair in the subsequence of the first *i* amino acids of the protein sequence. A one-to-one relationship is applicable to all 158 specified physicochemical properties and right circular cones. Therefore, 158 corresponding 3D graphical curves for each protein sequence can be obtained. In order to further convert it into a vector, we construct the *L*/*L* matrix of each curve, denoted *M*. In the matrix *M*, the element *M*_*ij*_ is the Euclidean distance between the coordinate *S*_*i*_ and the coordinate *S*_*j*_. The diagonal coordinate is marked as 0. Next, we calculate the eigenvalue of the real symmetric non-negative matrix and record its leading eigenvalue as the output in the *FV* vector.

### Modified Information Bottleneck to Variational Information Bottlenecks

In the original paper,^3^ the authors implemented information bottleneck as the feature extraction optimization. Simply put, the information bottleneck monitors the feature engineering process to maintain optimized latent representation. It keeps the ratio of relevant information to noise to the maximum. As a modified version of information bottlenecks, variational information bottleneck^16^ can effectively suppress irrelevant features learned from sequence encoding, and reduce the risk of overfitting, which is brought about by the huge number of parameters of the pretrained model.

**Figure 5:**
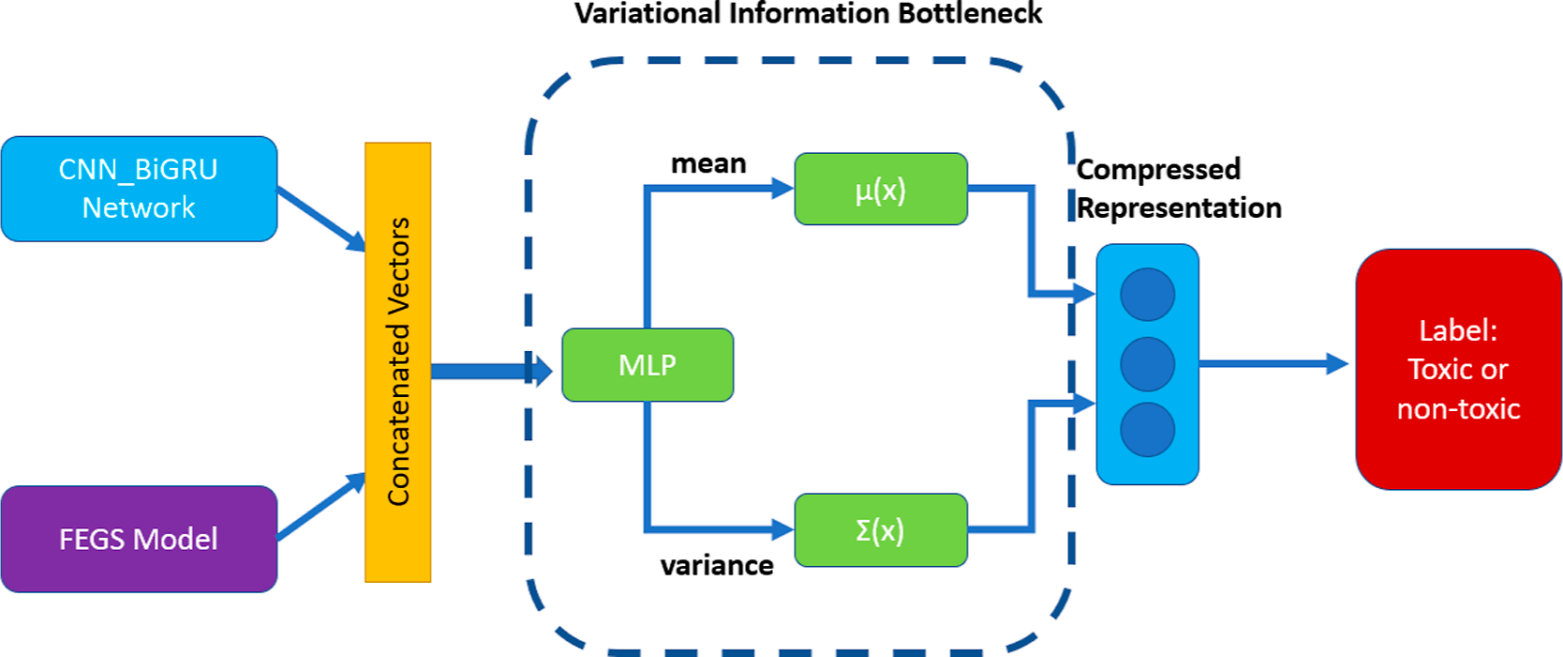
Revised architecture of modification design #5 (modify information bottleneck to variational information bottleneck).

Irrelevant information in variational information bottleneck is inhibited as regularization is implemented to training loss. This also helps to reduce the risk of overfitting when trained on a small amount of data. As illustrated in Figure 5, the string embedding from the pretrained model is converted to a latent representation *z*. Afterward, categorization results are totally based on the input of *z* since the *z*-value is chosen in accordance with the information bottleneck principle, which stipulates that the input should contain all the information necessary for the classification task. It is worth mentioning that the variational information bottleneck contributes heavily to the accuracy of the representation. Therefore, a singly useful feature could still be removed if it is unnecessary when in combination with other features.

For our study, the modification is made in the classification step. Before implementing information bottleneck schema to get the mean and covariance of latent feature vector *z*, we first add an extra MLP classifier to the concatenated sequence vectors. In this way, we get compressed sequence representations which could decrease the risk of overfitting.

### Evaluation Metrics

In order to compare the results, we adopted similar evaluation metrics used in the benchmark paper: accuracy, sensitivity, and specificity. Moreover, the F1-score and Mathew’s correlation coefficient (MCC) were proposed to overcome the trade-off between sensitivity and specificity. These metrics are described in previous bioinformatics papers^19,20^ as shown in the following equations. True positive (TP) represents the number of samples that detect toxicity as toxicity, false negative (FN) represents the number of samples that detect toxicity as nontoxicity, true negative (TN) represents the number of samples that detect nontoxicity as nontoxicity, and false positive (FP) represents the number of samples that detect nontoxicity as toxicity.
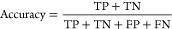
9
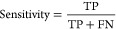
10
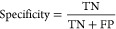
11
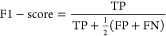
12
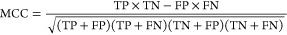
13We used the 10-fold cross-validation technique to evaluate the performance of the model. McNemar’s test^21^ was performed to test the significance among different models. After evaluating and selecting models, the final model was tested on the independent test set (testing data).

## Results and Discussion

### Performance Results of Different Modification Models

All performance results are listed in Table 2. As in the original paper,^3^ the modification models are evaluated using the same protein and peptide dataset. The assessment result shows that the first modification model shows slightly better performance in both protein and peptide datasets when compared to the original model on the peptide dataset. Specifically, the prediction accuracy increased by 0.41% on the protein dataset and 0.23% on the peptide dataset. We believe that there are two possible reasons for this result: one possible reason is that the improvement may not be obvious because a single self-attention layer may be unable to capture important details from multiple angles and levels. Another possible reason is that the self-attention layer establishes a larger global receptive field and introduces more global information, which may cause ambiguity in the CNN_BiGRU network structure, preventing better feature extraction.

**Table 2:** Summary of Model Performance on Protein and Peptide Datasets^a^

	protein			peptide		
optimization	Acc (%)	F1	MCC	Acc (%)	F1	MCC
ToxIBTL^3^	96.30	0.6775	0.6499	94.77	0.9428	0.9473
applied self-attention in BiGRU	96.71	0.7477	0.7242	95.00	0.9466	0.9497
replaced BiGRU with transformer (multihead attention)	95.96	0.6916	0.6645	87.21	0.8692	0.8648
applied channel attention in both the CNN and BiGRU	96.73	0.6745	0.6487	95.03	0.9553	0.9464
added numerical information to FEGS encoding	96.24	0.6813	0.6508	93.97	0.9402	0.9383
variational information bottleneck	97.38	0.7528	0.7310	94.90	0.9502	0.9483

a ToxIBTL: previous paper, Acc: accuracy, F1: F1-score, MCC: Matthews correlation coefficient, FEGS: feature extraction based on graphical and statistical features.

It is very surprising to see that although modification model #2 improved the efficiency, it did not achieve better prediction accuracy on both protein and peptide datasets compared to the reproduced model. The only possible reason is that the pretrained dataset (approximately 10,000 protein sequences) and the fine-tuned dataset (approximately 3000 protein sequences) are relatively small compared to the common transformer training dataset (more than 1,000,000 pieces of data) which prevents the transformer from reaching its full potential. Therefore, when the magnitude of the dataset remains unchanged, using the features extracted by BiGRU will achieve higher prediction accuracy than using the features extracted by the transformer.

From the test results, we can see that modification model #3 slightly improved the prediction accuracy and sensitivity compared to the reproduced model. Specifically, for the protein and peptide datasets, the prediction accuracy increased by 0.43 and 0.26%, respectively. Furthermore, the sensitivity of the peptide dataset is 96.20%. After making a comparison with the reproduced model, which only has a value of 94.80% in sensitivity, we drew the conclusion that the modification improves the ability to identify toxic peptides. In general, this design can be viewed as a relatively successful attempt at the peptide toxicity prediction task.

After making modifications to the FEGS model (modification model #4), we tested it while keeping the rest of the model unchanged. Its performance is the same as the original paper^3^ on the protein dataset. However, the highest accuracy rate on the peptide test set is only 93.97%, which decreased by 0.8% compared to the reproduced baseline model. Simultaneously, the model’s sensitivity on the test set is 93.1%, which is 1.7% lower than the baseline model.

A few key metrics are to be noted when comparing the modification model #5 with the reproduced model. On the pretrained protein dataset, the revised model achieved 97.38% prediction accuracy, which is 1.08% higher than 96.30%. Meanwhile, the best prediction accuracy on the fine-tuning peptide dataset is 94.90%, which is 0.27% greater than the baseline model’s 94.77%. Other metrics such as the F1-score (0.9502) and MCC (0.9483) are also greater than the original paper.^3^ Therefore, this modification model outperformed previous models in terms of all measurement metrics.

### Comprehensive Comparison

In this study, we briefly discuss five representative designs and analyze their performances on protein and peptide datasets, respectively. Out of the five modification designs, four modifications focused on sequence encoding to enhance the feature extraction capabilities of ToxIBTL,^3^ and the last one targeted an information bottleneck for better compression sequence representations. Table 2 is a summary of model performance on protein and peptide datasets.

Ray Tune hyperparameter search algorithms^22^ are utilized to perform scalable hyperparameter tuning, which efficiently optimize our model. To select the best combination of hyperparameters for the pretrained model and the fine-tuned model, we conduct at least four trials with 10 sets of hyperparameters per trial. All models converge within 500 epochs on the protein dataset and 150 epochs on the peptide dataset. Since some of the revised models (such as the transformer model) were relatively slower to converge with smaller CNN strides, the optimizer was changed from Adam to AdamW.

Compared to the original model, most of the revised models slightly improved the prediction accuracy on both the protein dataset and peptide dataset. On the protein dataset, the highest prediction accuracy (97.38%) comes from the revised model which included variational information bottlenecks. This performance means that our proposed model outperformed not only the ToxIBTL model^3^ but also other protein-based toxicity predictors (e.g., BLAST,^11^ InterProScan,^23^ HMMER,^24^ ClanTox,^6^ ToxinPred,^7^ and ToxDL^4^). To see the significant differences among methods, we performed a McNemar’s test^21^ to statistically test the differences between our results and the latest predictor—ToxIBTL.^3^ The statistical test then showed that our performance has been significantly improved in terms of F1-score and MCC compared to ToxIBTL performance. As for the peptide dataset, the model that included channel attention has the highest prediction accuracy (95.03%) and sensitivity (96.20%). Similarly, the peptide-based toxicity predictor was superior to previous studies including ToxIBTL,^3^ ClanTox,^6^ ToxinPred,^7^ and ATSE.^10^ Unfortunately, McNemar’s test did not tell any significant differences between our results and other predictors. However, a slight improvement in the peptide dataset and a significant improvement in the protein dataset has proven the efficiency of our method in this challenging problem.

**Figure 6:**
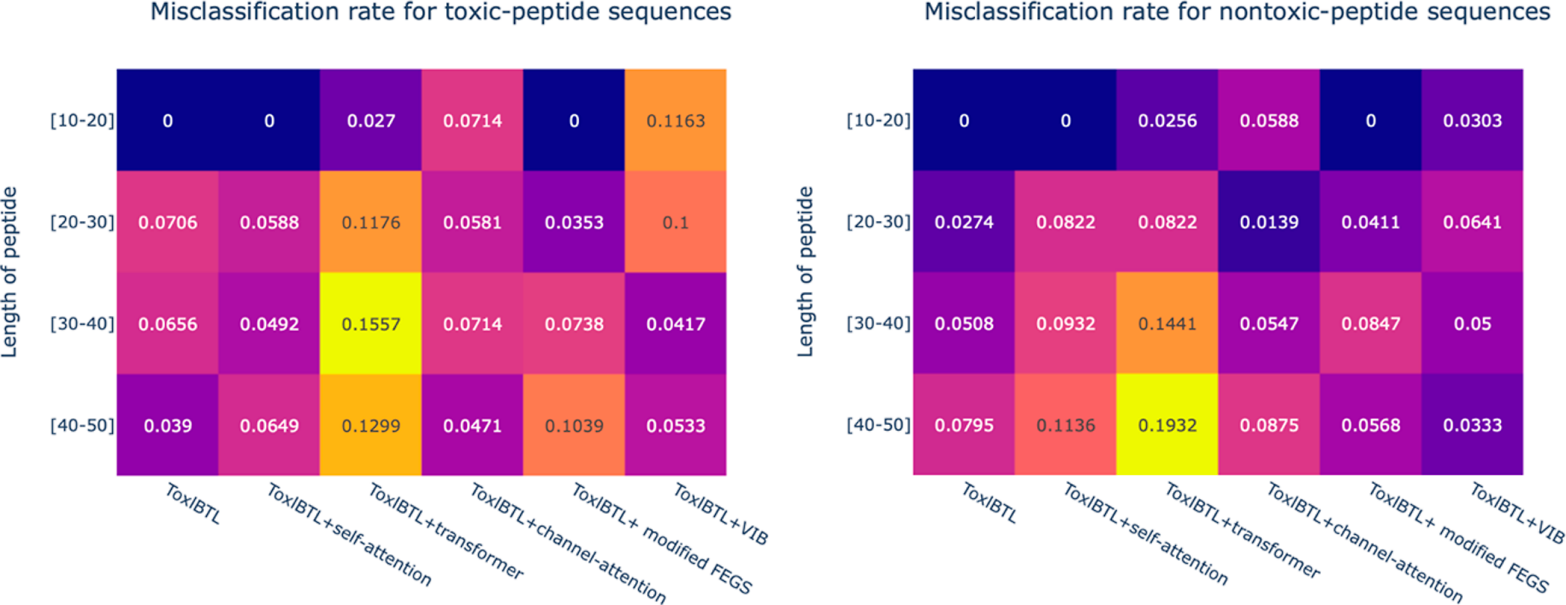
Heat map of misclassified peptide samples. (A) Toxic peptides and (B) nontoxic peptides.

In order to understand how peptide sequences of various lengths affect the classifiers in making the right predictions, we compare the rate of wrong predictions within different length intervals of ToxlBTL^3^ and our five modification designs, including ToxIBTL + self-attention, ToxIBTL + transformer, ToxIBTL + channel attention, ToxIBTL + modified FEGS, and ToxIBTL + VIB (variational information bottleneck) toward toxic peptides and harmless peptides in the test set. The visualization heat maps are shown in Figure 6. From the heat map (Figure 6A), we can see that almost all models show good classification performance, especially ToxlBTL + self-attention and ToxlBTL + channel attention. These models can keep the error rate lower than 10% in all length intervals. Furthermore, ToxlBTL + modified FEGS and ToxlBTL + VIB are also stable throughout a range of peptide lengths. The error rate in ToxlBTL + modified FEGS decreases, with the peptide length getting shorter, while the rule in ToxlBTL + VIB is quite the opposite: lower error rate in longer sequences. For the ToxlBTL + transformer, it has a relatively higher misclassification rate compared to other models and the error rate is higher on the longer peptides. For the heat map in Figure 6B, ToxlBTL + channel attention, ToxlBTL + modified FEGS, and ToxlBTL + VIB show better performance, which has a misclassification rate lower than 10% throughout different length intervals. ToxlBTL + transformer still shows a relatively higher misclassification rate on the longer length interval.

In general, all modifications are not biased toward toxic or harmless peptides and can give reliable predictions between toxic and harmless peptides across different length intervals. Hence, our designs are reliable and accurate predictors for predicting the toxicity of peptides of any length.

### Further Discussion and Limitations

Although our modification designs did not show obvious improvements in prediction accuracy compared to the reproduced baseline model, the contributions come from the following aspects: first, in our third modification design (adding channel attention to CNN and bidirectional GRU network), the sensitivity reaches 96.2%, which is 1.4% higher than the result we achieve in the reproduced baseline model. This means that the modification can lead to extraordinary performance in identifying toxic peptides. Second, the other four modification designs also showed relatively good performance with reference to other newly proposed methods, meanwhile adding novelty to peptide toxicity prediction. For example, in the second design [replacing bidirectional GRU with the transformer (based on multihead attention)], parallel computing is enabled. We anticipate that our study could lead to new thoughts and orientations in the peptide-related pharmacy domain.

With promising results achieved so far, we still have some space for improvement. First, we can customize a new protein and peptide dataset by searching sequences from other famous protein databases like Protein Data Bank (PDB)^25^ and The Structural Classification of Proteins^26^ and then reassess the reproduced model and our optimization, respectively. Second, we can add secondary structures to transcoding methods, such as Miyazawa energies and Micheletti potentials.^27^ Finally, we may also consider improving the model interpretability by adding additional interpretable deep learning feature analysis in the future.^28−30^

## Conclusions

Peptide-based drugs can only be discovered and developed with accurate identification of their toxicity. To facilitate the process, this study proposed five designs to improve the recent deep learning method, which refers to data in graphics, statistics, and evolution to get abundant features. The designs are mainly focused on two aspects: sequence encoding an information bottleneck. For sequence encoding, we attempted to add self-attention, multihead attention, and channel attention to enhance the feature extraction capabilities of the CNN_BiGRU framework. Meanwhile, we also incorporated numerical information into the FEGS encoding process to utilize the AAIndex1 and extract the physicochemical information more precisely. For information bottlenecks, we modified information bottlenecks to variational information bottlenecks for better calculation of compressed sequence representations for classification and to avoid overfitting. Finally, our optimized model outperformed the other sequence-based toxicity predictors at both the protein level and peptide level. Supporting Information and corresponding source codes for each modification design are available at https://github.com/khanhlee/toxicity-vib.
